# Small business owners' health and safety intentions: A cross-sectional survey

**DOI:** 10.1186/1476-069X-4-23

**Published:** 2005-10-21

**Authors:** Lisa M Brosseau, Shelby Yahui Li

**Affiliations:** 1 Division of Environmental Health Sciences, School of Public Health, University of Minnesota, 420 Delaware St SE, Minneapolis, Minnesota, 55455, USA; 2 Cardiac Rhythm Management, Medtronic, Inc., 1015 Gramsie Road, Shoreview, Minnesota, 55126, USA

## Abstract

**Background:**

Little is known about the variables underlying small business owners' behavioural intentions toward workplace health and safety. This project explores the relationship between three mediating variables (*Attitude Toward Safety*, *Subjective Norm and Perceived Behavioural Control*) and owners' *Intentions Toward Safety*, following the Theory of Planned Behaviour. We also investigate the role of beliefs underlying each mediating variable.

**Methods:**

Seven hundred businesses (5–50 employees) were randomly selected from 4084 eligible companies in a manufacturing business database (SIC codes 24 to 39). The 348 respondents are on average 51 yrs of age, 86% male, 96% white and have 2 to 4 years of post-secondary school.

**Results:**

All three mediator variables are significantly correlated with *Intentions Toward Safety*; *Attitude Toward Safety *shows the strongest correlation, which is confirmed by path analysis. Owners with higher attitudes toward safety have a higher probability of believing that improving workplace health and safety will make employees' healthier and happier, show that they care, increase employee productivity, lower workers' compensation costs, increase product quality and lower costs.

**Conclusion:**

These results suggest that interventions aimed at increasing owners' health and safety intentions (and thus, behaviours) should focus on demonstrating positive employee health and product quality outcomes.

## Background

Small businesses are an important sector of the United States economy. Nearly 98% of the 5.7 million U.S. businesses have fewer than 100 employees and account for 36% of all employment [1]. Ninety-three percent of the approximately 305,000 U.S. manufacturing firms have fewer than 100 employees [2].

Employees in small and medium-sized manufacturing businesses experience higher levels of work-related injuries and illnesses than employees in large businesses. For example, the 2003 incidence rates for non-fatal injuries and illnesses in the United States were highest in businesses with 50 to 249 employees in all economic sectors. In the manufacturing sector, the highest rates of non-fatal injuries and illnesses are experienced in durable goods manufacturing businesses with 11 to 249 employees [3].

Data on owners' behaviours toward health and safety in small businesses are limited and conflicting. In interviews, owners describe numerous barriers including limited resources, lack of in-house expertise, and production pressures [4,5]. One study found that insurers, quality assurance programs and regulatory agencies are important incentives to improve health and safety [5], while another found that owners do not trust government agencies or consultants and seek input on environmental improvements only from suppliers, other owners and customers [6]. Champoux and Brun found that most small business owners do not think that resources are a significant barrier to their improving health and safety. Only 37% of 223 owners of small businesses (fewer than 50 employees) thought cost was an important barrier to health and safety improvements [7].

Eakin et al. describe additional barriers, including owners' limited perspective about what can be accomplished, limited unionization and informal management structures [4]. Numerous investigators have shown that the incidence of injuries is lower in businesses that actively engage employees in decision making and joint labour-management safety committees [8-11]. However, Champoux and Brun found that fewer than 5% of small businesses had participatory safety programs [7].

A few investigators have studied the effectiveness of health and safety interventions in large and small businesses using randomized, controlled trials [5,12-14], but have been largely unsuccessful at bringing about significant changes in workplace health and safety. It has been conjectured that intervention activities may require more focused efforts targeted at motivating business owners to make improvements, in addition to changing the behaviour of employees [12,14].

This project was prompted by the need for more information about the factors that influence small business owners' intentions toward workplace health and safety. We selected the Theory of Planned Behaviour [15] to guide the development of a survey of owners' intentions. Behavioural intentions are measured as a surrogate for actual behaviour, which must be defined in terms of action, target, context and time. Three mediating variables – *Attitude Toward the Behaviour*, *Subjective Norm *and *Perceived Behavioural Control *– combine to influence a person's intentions toward the target behaviour. Three types of beliefs may indirectly contribute to *Behavioural Intention*: *Outcome Beliefs*, *Normative Beliefs *and *Control Beliefs*.

**Figure 1:**
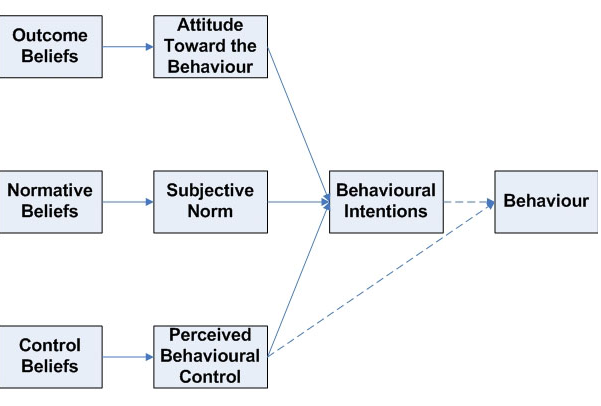
Theory of planned behaviour.

We used a modification (Figure 1) of the original model, eliminating the measurement of belief strength for each of the three belief constructs, because initial testing indicated that respondents did not understand the difference between these constructs. The specific behaviour is defined as: "In the next six months [time], how likely is it you will improve [action] workplace health and safety [target] in your business [context]."

The goals of this project are to 1) evaluate the association of the three mediating variables with owners' health and safety intentions and 2) investigate the role various beliefs play in formulating intentions. Insights from these relationships are expected to lead to the development of more effective interventions.

## Methods

### Survey Design

Following a protocol developed by Ajzen and Fishbein [16], we conducted open-ended telephone interviews with 16 small business owners in a variety of industries. Owners were asked to identify outcomes of their behaviour (what happens when you work on health and safety?), who influences the behaviour (who encourages or discourages you to work on health and safety?), and barriers and supports for the behaviour (what helps or hinders you to work on health and safety?). Answers to these three questions were categorized and the most frequent responses were used to develop survey items for specific outcome, normative and control beliefs, respectively.

A draft survey was developed and reviewed for face validity by experts in health and safety and small business assistance. Two pilot studies were conducted, each of which involved initial and follow-up (with a $2 incentive) mailings to 120 independent manufacturing businesses with 5 to 50 employees in Minnesota. The data from these surveys received a preliminary analysis to determine if any changes in survey design were necessary. Only minor changes in wording and organization were made before the main study.

In the main study, the survey was mailed to 700 owners of small manufacturing businesses in Minnesota, with a second mailing, including a $2 incentive, three weeks later.

### Study Population

Eligible businesses were drawn from the Manufacturers' News, Inc. manufacturing businesses database (2001) for Minnesota, which included all businesses in SIC codes 24 to 39. The final set included 4084 businesses with 5 to 50 employees established after 1998 (in the database at least 3 years) without parent companies (independent businesses). Seven hundred businesses were drawn randomly from the set; respondents to the pilot study surveys were not included in the main trial.

In all, there were 348 respondents, a 49.7% response rate. Respondents are on average 51 yrs of age (standard deviation = 10.22), 86% male, and 96% white. They have a mean maximum education level between 2 and 4 years of post-secondary school. On average, businesses have been in operation 30 years; respondents have worked in the industry for 23 years and owned their business 16 years. Companies have a mean of 14 production and 22 total employees. Sixty-six percent indicate they are the president, 49% are owners, and 22% are managers (respondents could indicate one or more positions).

### Survey Content

Each variable is measured by one or more items in the survey (Table 1). The score for a question is the sum of scores for each item in the question. Questions about intentions, attitudes, subjective norm, outcome beliefs and normative beliefs were operationalised as described by Ajzen and Fishbein [16]. Questions about behavioural control and control beliefs used a format described by Fishbein [15].

**Table 1:** Description of variables and basic statistics

Variable Name	Number of Items	Mean* (Standard Deviation)	Range*	N
(Y) Intentions Toward Safety	9	19 (8.2)	9–45	330
(Z_1_) Attitude Toward Safety	3	6.4 (2.5)	3–15	326
(Z_2_) Subjective Norm	1	1.9 (0.8)	1–5	345
(Z_3_) Perceived Behavioural Control	1	2.6 (1.0)	1–5	344
(X_1_) Outcome Beliefs	11	32 (5.4)	15–52	339
(X_2_) Normative Beliefs	5	16 (4.5)	5–25	343
(X_3_) Control Beliefs	4	9.5 (3.0)	4–17	344

* Lower scores correspond to more positive responses.

### Intentions

*Intentions Toward Safety *is measured by the sum of nine questions asking "In the next six months, how likely is it you will [take a specific action]?" (measured on a 5-pt scale ranging from 1 = "very likely" to 5 = "very unlikely"). The specific actions were:

• Talk to employees about health and safety rules

• Reward employees for following safe work rules

• Wear safety equipment when enter the work area

• Walk through business and identify safety hazards

• Check that employees are wearing safety equipment

• Make sure access is clear to exits and extinguishers

• Talk to employees about the hazards of their job

• Train employees to handle emergencies

• Ask employees for recommendations on safer ways to do their work

In many cases, complex behaviours cannot be measured by a single action (e.g. weight loss involves both dieting and exercise behaviours). In such cases, a behavioural index comprised of several individual behaviours will provide a better measure [16]. Improving workplace health and safety is certainly a complex behaviour, but there is no accepted set of actions that define such behaviour for a business owner. Some health and safety behaviours are one-time, programmatic actions, while others are activities that must take place on a regular basis.

We focused on health and safety activities that owners should conduct on a regular basis, but did not specifically define "regularly," because it will vary by activity, industry, number of employees, etc. We did not ask about specific programs or hazards, because owners were located in a broad range of manufacturing industries.

The topic of health and safety behaviour has received very little formal research or validation. We drew our list of actions from those developed by practitioners and regulators to describe "good" health and safety. The best examples of these are found in recognition programs, such as the United States Occupational Safety and Health Administration's Voluntary Protection Program (VPP) [17] and in health and safety management systems proposed by individuals and organizations [18-20]. Several occupational health professionals with small business experience and a range of perspectives (e.g. consulting, business assistance, regulatory) were asked to review this set of actions for face validity.

### Attitude

*Attitude Toward Safety *is measured by the sum of three questions about the importance, necessity and convenience of improving health and safety in the next six months (scored on a 5-pt scale; for example: 1 = "very convenient"; "somewhat convenient"; "neither"; "somewhat inconvenient"; 5 = "very inconvenient").

With input from small business owners and health and safety professionals, we selected these three as the most relevant to health and safety behaviour using guidelines developed by Osgood et al. [21].

### Subjective Norm

*Subjective Norm *is measured by agreement (1 = strongly agree; 5 = strongly disagree) with "Most people important to me think I should improve health and safety in my business in the next six months."

### Perceived Behavioural Control

*Perceived Behavioural Control *is measured by how easy it will be for owners to improve health and safety in their business in the next six months (1 = very easy; 5 = very difficult).

### Outcome Beliefs

*Outcome Beliefs *are measured by summing answers to questions about the likelihood of eleven outcomes (5-pt scale; 1 = "very likely" to 5 = "very unlikely" scale):

1. Make employees happier

2. Make employees healthier

3. Increase costs

4. Increase employees' productivity

5. Cause employees to complain

6. Show that I care about employees

7. Cut into profits

8. Lower workers' compensation costs

9. Take too much time

10. Increase the quality of products

11. Lower the business' productivity

These eleven outcome beliefs were identified from the most frequent responses in our open-ended interviews with small business owners. Ajzen and Fishbein discourage the selection of outcome beliefs not derived from interviews with the target population [16].

### Normative Beliefs

*Normative Beliefs *are measured by summing the responses to questions about the influence of five groups on owners' behaviour (5-pt scale, 1 = "strongly agree" to 5 = "strongly disagree"):

1. My employees think I should improve safety in my business.

2. My workers' compensation company...

3. Government agencies...

4. My customers...

5. My vendors or suppliers...

As with outcome beliefs, these five normative beliefs were selected from the most frequent responses of interviewed owners.

### Control Beliefs

*Control Beliefs *are measured by summing responses to four questions (5-pt scale; 1 = "strongly agree" to 5 = "strongly disagree"):

1. I have enough resources available for improving safety in my business.

2. I am well-informed about how to improve safety in my business.

3. My employees are supportive of my efforts to improve safety in my business.

4. I have enough time to improve safety in my shop.

These four beliefs were derived from the most frequent responses obtained in owner interviews.

## Results

Correlation analysis, linear and logistic regression and structural equation modelling techniques were used to explore relationships among the variables of interest. Negative outcome beliefs (#3, 5, 7, 9 and 11 shown above) were re-coded to ensure that all outcome beliefs were evaluated in the positive direction.

Pearson's correlation analysis was first applied to examine the relationship between the response variable, *Intentions Toward Safety*, and the three main covariates: *Attitude toward Safety*, *Subjective Norm*, and *Perceived Behavioural Control *(Table 2). The response variable was transformed to the log scale to satisfy the normality assumption. All three variables were significantly correlated with *Intentions Toward Safety*; correlation with *Attitude Toward Safety *was the strongest, ρ = 0.56 (p < 0.0001).

**Table 2:** Correlation between the response variable (Log(Intentions Toward Safety)) and covariates*

Variable	Correlation Coefficient	p-value	N
Attitude Toward Safety	0.559	<0.0001	326
Subjective Norm	0.370	<0.0001	345
Perceived Behavioural Control	0.297	<0.0001	344

* The variable "Intentions Toward Safety" is in the log scale to satisfy the normality assumption.

We also explored correlations between the covariates (Table 3). The correlations between *Attitude Toward Safety *vs. *Outcome Beliefs *and between *Perceived Behavioural Control *vs. *Control Beliefs *are moderate (ρ = 0.46 and 0.42, respectively) (p < 0.05). The correlation of *Subjective Norm *with *Normative Beliefs *is weak (ρ = 0.10; p = 0.06).

**Table 3:** Correlation between covariates

Covariates	Correlation Coefficient	p-value	N
Attitude Toward Safety vs. Outcome Beliefs	0.459	<0.0001	326
Perceived Behavioural Control vs. Control Beliefs	0.425	<0.0001	344
Subjective Norm vs. Normative Beliefs	0.101	0.06	345
Attitude Toward Safety vs. Perceived Behavioural Control	0.449	<0.0001	326
Attitude Toward Safety vs. Subjective Norm	0.384	<0.0001	326
Subjective Norm vs. Perceived Behavioural Control	0.271	<0.0001	345

Finally, six of the eleven outcome beliefs are moderately correlated with owners' *Attitude Toward Safety *(p < 0.05) (Table 4):

**Table 4:** Correlations between outcome beliefs and *Attitude Toward Safety*

Outcome Belief	Correlation Coefficient	p-value
Make employees happier	0.489	<0.0001
Make employees healthier	0.538	<0.0001
Decrease costs*	-0.092	0.10
Increase employee productivity	0.385	<0.0001
Not cause employee complaints*	-0.112	0.04
Show that I care about employees	0.422	<0.0001
Not cut into profits*	-0.048	0.38
Lower workers' compensation costs	0.282	<0.0001
Not take too much time*	0.165	0.003
Increase product quality	0.331	<0.0001
Raise business productivity*	0.006	0.91

* Wording has been changed from the original to reflect re-coding.

• Make employees healthier

• Make employees happier

• Increase employee productivity

• Show that I care about employees

• Lower workers' compensation costs

• Increase product quality

A total number of 300 surveys were available for structural equation modelling (i.e. no missing responses). Path analysis using AMOS 5.0.1 (SPSS Inc., 2003) was used to fit the following multiple regression equations simultaneously:

*Y *= *β*_1_*Z*_1 _+ *β*_2_*Z*_2 _+ *β*_3_*Z*_3 _+ *D *

*Z*_1 _= *γ*_11_*X*_1 _+ *D*_1_

*Z*_2 _= *γ*_21_*X*_2 _+ *D*_2_

*Z*_3 _= *γ*_31_*X*_3 _+ *D*_3_

where

Y = log (*Intentions Toward Safety*)

*Z*_1 _= *Attitude toward Safety*

*Z*_2 _= *Subjective Norm*

*Z*_3 _= *Perceived Behavioural Control*

*X*_1 _= *Outcome Beliefs*

*X*_2 _= *Normative Beliefs*

*X*_3 _= *Control Beliefs*

*D*_1_, *D*_2_, *D*_3 _and *D *are *disturbances *and β's and *γ*'s are regression coefficients.

Although sample size does not contribute to the identifiability of a path model, it does contribute to the precision of path analysis estimates. There were 7 main variables observed for this study, therefore a maximum of 28 parameters could be estimated. Using the rule that N/P should be greater than 10, where N = number of subjects, P = number of parameters, we found that the statistical stability of the results from this model should be acceptable [22].

**Figure 2:**
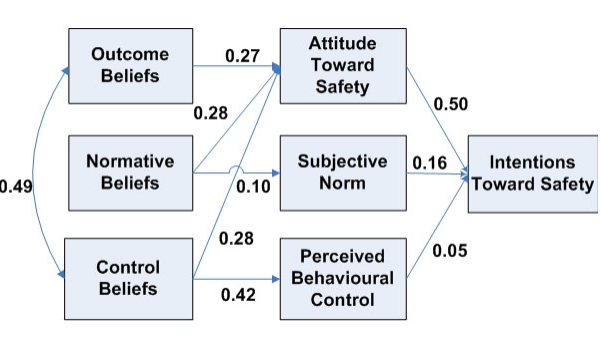
Path analysis results.

A fitted path model is shown in Figure 2. Arrows symbolize direct effects. The values associated with each path are standardized regression coefficients (weights), which represent the amount of standard deviation change in a dependent or mediating variable given a standard deviation change in the corresponding predicting variable while holding other variables constant. Since we observed significant correlations between the belief variables and *Attitude Toward Safety *during the model modification process, the direct effects of *Normative Beliefs *and *Control Beliefs *on *Attitude Toward Safety *were added to the path model.

The path analysis confirmed the significant effect of *Attitude Toward Safety *on *Intentions Toward Safety *(weight = 0.50). *Attitude Toward Safety *was equally moderately affected by *Outcome Beliefs*, *Normative Beliefs *and *Control Beliefs *(weights of 0.27, 0.28 and 0.28, respectively). The direct effects of *Subjective Norm *and *Perceived Behavioural Control *on *Intentions Toward Safety *were not strong (weights of 0.16 and 0.05, respectively). The link between *Normative Beliefs *and *Subjective Norm *was also weak (weight of 0.10). *Outcome *and *Control Beliefs *were also strongly associated (weight = 0.49).

We also used logistic regression to assess various relationships between the predictor variables and *Intentions Toward Safety*. Using a technique proposed by Ajzen and Fishbein [16], *Intentions Toward Safety *was dichotomized at its median (a better approximation of the center of a skewed distribution) into two groups: owners with high intentions and those with low intentions. The probability of having high intentions was regressed on *Attitude Toward Safety*, *Perceived Behavioural Control *and *Subjective Norm*. Results showed that *Attitude Toward Safety *(p < 0.0001) and *Perceived Behavioural Control *(p = 0.0153) were both significantly associated with *Intentions Toward Safety *(Table 5). Age was also considered, but did not have a significant effect on the outcome.

**Table 5:** Odds ratio estimates for covariates on dichotomized response variable (*Intentions Toward Safety*)

Covariate	Odds Ratio	95% Confidence Limits
Attitude Toward Safety	1.4	1.2, 1.6
Perceived Behavioural Control	1.3	1.0, 1.7
Subjective Norm	1.7	1.2, 2.5

The largest effect in the path model on log(*Intentions Toward Safety*) comes from *Attitude Toward Safety*. Therefore, we further explored which specific beliefs might be associated with business owners' safety attitudes. *Attitude toward Safety *was dichotomized into "high" vs. "low" at its median. Logistic regression was used to model the probability of having high *Attitude Toward Safety*. We first examined the association between the eleven outcome beliefs and *Attitude Toward Safety *(Table 6). Three outcome beliefs showed a high probability of being associated with higher attitudes toward improving safety:

**Table 6:** Odds ratio estimates for covariates on dichotomized response variable (*Attitude Toward Safety*)*

Outcome Belief	Odds Ratio	95% Confidence Limits
Make employees healthier	2.02	1.51, 2.70
Lower costs	1.50	1.06, 2.11
Show that I care	1.39	1.08, 1.80

* Stepwise selection of variables. Only variables with significant effects are shown.

1. Make employees healthier

2. Lower costs

3. Show that I care

All of the specific beliefs (*Outcome*, *Normative *and *Control*) were then added to the model. One outcome belief (make employees happy), one normative belief (a workers' compensation company that thinks owners should improve health and safety) and two control beliefs (being well-informed and having supportive employees) had a high and significant probability of being associated with higher attitudes toward improving safety.

## Discussion

Results from the correlation and path analyses suggest that business owners' intentions toward improving safety are most strongly associated with their attitude toward safety. While neither subjective norm nor behavioural control are predictors of owners' intentions, they are moderately correlated with owners' attitudes toward safety. In addition, outcome and control beliefs are both moderately correlated with attitudes.

These results suggest that it is owners' attitudes that most strongly influence their intentions (and thus behaviours) to improve employee health and safety. A small set of outcome beliefs is associated with higher attitudes. Correlation and logistic regression analyses suggest that this set includes beliefs that improving health and safety will make employees healthier and show that I care. Other outcome beliefs (occurring in at least one of the analyses) that may influence health and safety attitudes include:

• Make employees happier

• Increase employee productivity

• Lower workers' compensation costs

• Increase product quality

• Lower costs

Knowledge, supportive employees, and an influential workers' compensation company are also associated with a more positive attitude toward workplace health and safety.

These results suggest that interventions should be aimed at increasing owners' expectations about the positive outcomes of improving health and safety and building more positive interactions between employees and owners. Demonstrating that business productivity and employee well-being can be enhanced by improvements in health and safety may also lead to higher attitudes. And workers' compensation companies may play an important role in raising attitudes (and thus, intentions) toward workplace health and safety.

There is general consensus that high levels of workplace health and safety require both management commitment and employee involvement [8,9], which are measured by observing the presence of specific activities, programs, systems, and policies. We found no systematic study of owners' or managers' beliefs and attitudes or their relation to intentions and behaviours. The majority of safety behaviour research has focused on employees' opinions about workplace safety, usually termed "safety climate," compared to safety behaviours or injury rates.

A few investigators have explored the relationship between managers' and employees' beliefs about workplace health and safety. For example, Rabin et al. found that employees are more likely to report receiving information about workplace hazards when managers hold more positive outcome beliefs and have more confidence about helping others [23]. Parker et al. found that employee self-reported safe behaviour is associated longitudinally with supportive supervision, job autonomy and the quality of communication about their job [24]. Both suggest that interventions be aimed at improving supervisors' communication with employees about safety. These corroborate our findings that the relationship between owners and employees is important to the development of high intentions toward health and safety among owners.

### Study Limitations

Given a single study, it is seldom appropriate to infer causality from the results of statistical analyses, including path analysis. When the variables are concurrently measured, researchers have to make a very clear rationale for specifying the direction of causal effects since ultimately path analysis deals with correlation, not causation [25]. However, the path analysis shows that the data we collected were mostly consistent with the hypothesized model. Cause and effect can be established through intervention trials in which subjects undergo the same experience except for the single facet of interest [25].

Non-response bias is always a concern with survey studies, since owners not responding may differ in some important way from the respondents. However, a 50% response rate is much higher than normally encountered in this population [26]. Government agencies and business associations generally encounter very low response rates (20–30%) unless much more intensive survey methods (e.g. multiple telephone calls) are used. Certainly, non-response should be considered when interpreting these data. However, we believe that these results are still important and relevant to designing interventions in small businesses.

This study relies on self-reported intentions to improve health and safety, which are not validated by observations of behaviour. In many cases intentions have been shown to be correlated with actual behaviour [16]. Resources were not available in this study to observe or measure owners' behaviour.

## Conclusion

The results of this study suggest that small business owners' intentions toward improving workplace health and safety are primarily influenced by their attitudes. Owners' outcome, normative and control beliefs all contribute to their attitudes toward workplace health and safety. Subjective norm and perceived behavioural control do not have any significant impact on small business owners' behavioural intentions toward workplace health.

Interventions aimed at these underlying beliefs, particularly those shown to be most highly associated with high-intentioned owners, may be successful in bringing about improvements in attitudes, intentions and behaviour. Raising owners' expectations about positive employee health and business productivity outcomes may lead to long-term improvements in their attitudes, intentions and behaviour toward workplace health and safety.

## Competing interests

The author(s) declare they have no competing interests.

## Authors' contributions

LMB designed and carried out the survey studies, participated in the data analysis and drafted the manuscript. SYL performed the statistical analysis and helped draft the manuscript. Both authors read and approved the final manuscript.
